# 3,5-Bis(ethoxy­carbon­yl)-2,6-dimethyl-1,4-dihydro­pyridine-4-carboxylic acid

**DOI:** 10.1107/S1600536809024945

**Published:** 2009-07-04

**Authors:** De-Hong Wu, Ling Hu

**Affiliations:** a Ordered Matter Science Research Center, College of Chemistry and Chemical Engineering, School of Materials Science and Engineering, Southeast University, Nanjing 210096, People’s Republic of China

## Abstract

The title mol­ecule, C_14_H_19_NO_6_, was synthesized by the reaction of glyoxylic acid, ethyl acetoacetate and NH_4_HCO_3_. In the crystal structure, the dihydro­pyridine ring adopts an asymmetric boat-type conformation with the C atom bearing the carboxyl group showing a signficantly larger deviation [0.325 (2) Å] from the base plane then the N atom [0.137 (2) Å]. One of the ethyl groups is disordered over two positions with occupancies of 0.741 (10) and 0.259 (10). The crystal is stabilized by strong inter­molecular hydrogen bonds. N—H⋯O inter­actions form infinite chains in the *a* direction. O—H⋯O hydrogen bonds form typical carboxylic acid dimers, which link the N—H⋯O chains into a ladder-type double chain.

## Related literature

For the electrophysiological activity of 1,4-dipyridine derivatives, see: Fleckenstein (1977 ▶); Cutshall *et al.* (2002 ▶). For their biological activity, see: Triggle *et al.* (1980 ▶); Fossheim *et al.* (1982 ▶); Heinrich *et al.* (2004 ▶); Henry (2004 ▶).
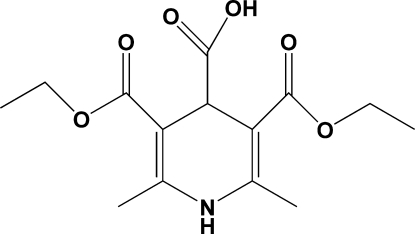


## Experimental

### 

#### Crystal data

C_14_H_19_NO_6_
*M* *_r_* = 297.30
Triclinic, 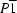

*a* = 7.445 (4) Å
*b* = 9.864 (5) Å
*c* = 11.908 (2) Å
α = 104.10 (3)°
β = 97.808 (9)°
γ = 111.658 (10)°
*V* = 763.2 (6) Å^3^
*Z* = 2
Mo *K*α radiation
μ = 0.10 mm^−1^
*T* = 291 K
0.36 × 0.30 × 0.28 mm

#### Data collection

Rigaku SCXmini diffractometer
Absorption correction: multi-scan (*CrystalClear*; Rigaku, 2005 ▶) *T*_min_ = 0.960, *T*_max_ = 0.970
6950 measured reflections
2969 independent reflections
2241 reflections with *I* > 2σ(*I*)
*R*_int_ = 0.029

#### Refinement

*R*[*F* ^2^ > 2σ(*F* ^2^)] = 0.063
*wR*(*F* ^2^) = 0.197
*S* = 1.08
2969 reflections
199 parameters
2 restraints
H-atom parameters constrained
Δρ_max_ = 0.29 e Å^−3^
Δρ_min_ = −0.24 e Å^−3^

### 

Data collection: *CrystalClear* (Rigaku, 2005 ▶); cell refinement: *CrystalClear*; data reduction: *CrystalClear*; program(s) used to solve structure: *SHELXS97* (Sheldrick, 2008 ▶); program(s) used to refine structure: *SHELXL97* (Sheldrick, 2008 ▶); molecular graphics: *SHELXTL* (Sheldrick, 2008 ▶); software used to prepare material for publication: *SHELXTL*.

## Supplementary Material

Crystal structure: contains datablocks I, New_Global_Publ_Block. DOI: 10.1107/S1600536809024945/kj2129sup1.cif

Structure factors: contains datablocks I. DOI: 10.1107/S1600536809024945/kj2129Isup2.hkl

Additional supplementary materials:  crystallographic information; 3D view; checkCIF report

## Figures and Tables

**Table 1:** Hydrogen-bond geometry (Å, °)

*D*—H⋯*A*	*D*—H	H⋯*A*	*D*⋯*A*	*D*—H⋯*A*
N1—H1*D*⋯O5^i^	0.86	2.17	3.018 (2)	167
O2—H2*A*⋯O1^ii^	0.82	1.82	2.641 (2)	176

Symmetry codes: (i) 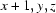; (ii) 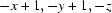.

**Fig. 1.**
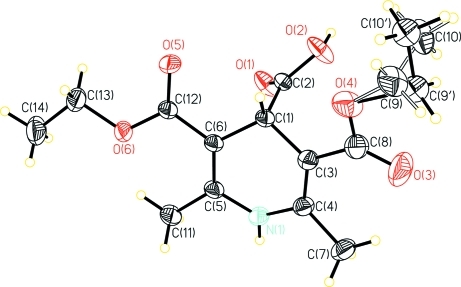
The molecular structure of the title compound, showing the atomic numbering scheme with 30% probability displacement ellipsoids. All disorder components are shown.

**Fig. 2.**
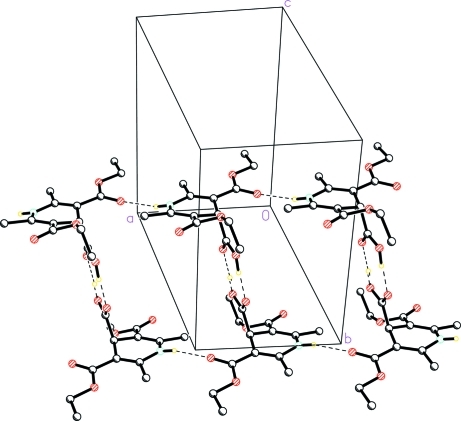
The crystal packing of the title compound, viewed down the b axis, showing chains along the [100] direction. Hydrogen bonds are shown as dashed lines. The hydrogen atoms except for H1D and H2D are omitted. Only major disorder components are shown.

## supplementary crystallographic information

### Comment

The development of new methods for the synthesis of 1,4-dipyridine derivatives is a motive for the current research because of their presence in numerous natural products along with a wide spectrum of their electrophysiological activities (Fleckenstein, 1977; Cutshall *et al.*, 2002). Pyridine 1,4-derivatives and their complexes have been studied for their fungicidal and antibacterial effects, antiviral drugs, as well as calcium antagonists (Triggle *et al.*, 1980; Fossheim *et al.*, 1982; Heinrich *et al.*, 2004; Henry, 2004).

Here we report the structure of 3,5-di(ethoxycarbonyl)-1,4-dihydro-2,6-dimethylpyridine-4- carboxylic acid (Fig. 1). In the crystal structure, the dihydropyridine ring adopts a asymmetric boat-type conformation with C1 showing a signficantly larger deviation from the base plane C3/C4/C5/C6 [0.325 (2) Å] then N1 [0.137 (2) Å]. The ethyl group labeled by C9 and C10 is disordered over two positions with occupancies of 0.741 (10) and 0.259 (10). The crystal is stabilized by strong intermolecular hydrogen bonds (Table 1). Interactions of type N—H···O form infinite chains in the *a*-direction. The O—H···O hydrogen bonds form typical carboxylic acid dimers which link the N—H···O chains into a ladder-type double chain (Fig. 2).

### Experimental

Glyoxylic acid (50% in water, 6 mmol), ethyl acetoacetate (12 mmol) and NH_4_HCO_3_ (6 mmol) were mixed in a 50 ml flask. After the mixture had been stirred for 6 h at 293 K, the crude product was obtained with yield of 65%. Single crystals suitable for X-ray structure analysis were obtained by the slow evaporation of a dimethylformamide solution in air.

### Refinement

H atoms, with exception of H2D bonded to O2, were placed on calculated positions (N—H = 0.86 Å; C—H = 0.96–0.98 Å for C*sp^2^* and C*sp^3^* atoms, respectively), assigned fixed *U*_iso_ values [*U*_iso_ = 1.2 *U*eq(C*sp^2^*/N) and 1.5 *U*eq(C*sp^3^*)] and allowed to ride. H2D was found with O—H = 0.97 Å in the difference electron density map. The ethyl group labeled by C9 and C10 is disordered over two positions with occupancies of 0.741 (10) and 0.259 (10), and all disordered atoms were subjected to a rigid bond restraint. The minor disorder component was refined with isotropic displacement parameters.

### Crystal data

**Table:**

C_14_H_19_NO_6_	*Z* = 2
*M**_r_* = 297.30	*F*(000) = 316
Triclinic, *P*1	*D*_x_ = 1.294 Mg m^−^^3^
Hall symbol: -P 1	Mo *K*α radiation, λ = 0.71073 Å
*a* = 7.445 (4) Å	Cell parameters from 2034 reflections
*b* = 9.864 (5) Å	θ = 2.3–27.5°
*c* = 11.908 (2) Å	µ = 0.10 mm^−^^1^
α = 104.10 (3)°	*T* = 291 K
β = 97.808 (9)°	Block, colourless
γ = 111.658 (10)°	0.36 × 0.30 × 0.28 mm
*V* = 763.2 (6) Å^3^	

### Data collection

**Table:**

Rigaku SCXmini diffractometer	2969 independent reflections
Radiation source: fine-focus sealed tube	2241 reflections with *I* > 2σ(*I*)
graphite	*R*_int_ = 0.029
Detector resolution: 13.6612 pixels mm^-1^	θ_max_ = 26.0°, θ_min_ = 3.0°
ω scans	*h* = −9→9
Absorption correction: multi-scan (*CrystalClear*; Rigaku, 2005)	*k* = −12→12
*T*_min_ = 0.960, *T*_max_ = 0.970	*l* = −14→14
6950 measured reflections	

### Refinement

**Table:**

Refinement on *F*^2^	Primary atom site location: structure-invariant direct methods
Least-squares matrix: full	Secondary atom site location: difference Fourier map
*R*[*F*^2^ > 2σ(*F*^2^)] = 0.063	Hydrogen site location: inferred from neighbouring sites
*wR*(*F*^2^) = 0.197	H-atom parameters constrained
*S* = 1.08	*w* = 1/[σ^2^(*F*_o_^2^) + (0.115*P*)^2^ + 0.1066*P*] where *P* = (*F*_o_^2^ + 2*F*_c_^2^)/3
2969 reflections	(Δ/σ)_max_ < 0.001
199 parameters	Δρ_max_ = 0.29 e Å^−^^3^
2 restraints	Δρ_min_ = −0.24 e Å^−^^3^

### Special details

**Table:**

Experimental. 1H NMR (DMSO-d6, p.p.m.): δ 1.17 (t, J = 7.0 Hz, 6H, CH2CH3), 2.21 (s, 6H, Me), 4.07 (m, J = 7.0 Hz, 4H, CH2CH3), 4.58 (s, 1H, CH), 8.84 (s, 1H, NH), 11.89 (S, 1H, OH). 13 C NMR (DMSO-d6, p.p.m.): δ 14.72 (CH2CH3), 18.39 (CH3), 39.71 (CH in dihydropyridine ring), 59.55 (CH2), 97.68, 146.27 (quaternary C in dihydropyridine ring), 167.33 (CO), 175.02 (COOH).
Geometry. All e.s.d.'s (except the e.s.d. in the dihedral angle between two l.s. planes) are estimated using the full covariance matrix. The cell e.s.d.'s are taken into account individually in the estimation of e.s.d.'s in distances, angles and torsion angles; correlations between e.s.d.'s in cell parameters are only used when they are defined by crystal symmetry. An approximate (isotropic) treatment of cell e.s.d.'s is used for estimating e.s.d.'s involving l.s. planes.
Refinement. Refinement of *F*^2^ against ALL reflections. The weighted *R*-factor *wR* and goodness of fit *S* are based on *F*^2^, conventional *R*-factors *R* are based on *F*, with *F* set to zero for negative *F*^2^. The threshold expression of *F*^2^ > σ(*F*^2^) is used only for calculating *R*-factors(gt) *etc*. and is not relevant to the choice of reflections for refinement. *R*-factors based on *F*^2^ are statistically about twice as large as those based on *F*, and *R*- factors based on ALL data will be even larger.

### Fractional atomic coordinates and isotropic or equivalent isotropic displacement parameters (Å^2^)

**Table:**

	*x*	*y*	*z*	*U*_iso_*/*U*_eq_	Occ. (<1)
C1	0.5752 (3)	0.3833 (2)	0.24556 (18)	0.0403 (5)	
H1A	0.4891	0.4029	0.2968	0.048*	
C2	0.5338 (3)	0.4308 (2)	0.13568 (18)	0.0418 (5)	
C3	0.7927 (3)	0.4825 (2)	0.31506 (19)	0.0471 (5)	
C4	0.9329 (3)	0.4276 (3)	0.29898 (19)	0.0482 (5)	
C5	0.6783 (3)	0.1670 (2)	0.19748 (19)	0.0447 (5)	
C6	0.5295 (3)	0.2141 (2)	0.21075 (17)	0.0403 (5)	
C7	1.1547 (3)	0.5129 (3)	0.3518 (3)	0.0700 (7)	
H7A	1.1858	0.6168	0.3980	0.105*	
H7B	1.2223	0.5136	0.2887	0.105*	
H7C	1.1974	0.4627	0.4026	0.105*	
C8	0.8460 (4)	0.6388 (3)	0.3946 (2)	0.0691 (7)	
C9	0.7022 (9)	0.7955 (6)	0.5064 (4)	0.0796 (16)	0.742 (10)
H9A	0.8340	0.8514	0.5609	0.096*	0.742 (10)
H9B	0.6041	0.7752	0.5532	0.096*	0.742 (10)
C10	0.6670 (9)	0.8858 (5)	0.4323 (6)	0.109 (2)	0.742 (10)
H10D	0.6721	0.9802	0.4824	0.164*	0.742 (10)
H10E	0.5379	0.8281	0.3771	0.164*	0.742 (10)
H10F	0.7678	0.9085	0.3887	0.164*	0.742 (10)
C11	0.6588 (4)	0.0046 (3)	0.1497 (3)	0.0639 (7)	
H11A	0.5206	−0.0659	0.1283	0.096*	
H11B	0.7320	−0.0178	0.2100	0.096*	
H11C	0.7114	−0.0059	0.0804	0.096*	
C12	0.3169 (3)	0.1123 (2)	0.18269 (19)	0.0439 (5)	
C13	0.0631 (4)	−0.1451 (3)	0.1230 (3)	0.0734 (8)	
H13A	−0.0118	−0.1280	0.0588	0.088*	
H13B	0.0062	−0.1315	0.1913	0.088*	
C14	0.0555 (5)	−0.3023 (3)	0.0839 (4)	0.0916 (11)	
H14A	−0.0808	−0.3768	0.0618	0.137*	
H14B	0.1301	−0.3177	0.1482	0.137*	
H14C	0.1121	−0.3142	0.0164	0.137*	
C9'	0.7854 (18)	0.8432 (14)	0.4818 (12)	0.065 (4)*	0.258 (10)
H9'A	0.8895	0.8831	0.5551	0.078*	0.258 (10)
H9'B	0.8364	0.8910	0.4238	0.078*	0.258 (10)
C10'	0.5997 (16)	0.8597 (14)	0.5033 (11)	0.070 (4)*	0.258 (10)
H10A	0.6312	0.9662	0.5417	0.105*	0.258 (10)
H10B	0.5459	0.8009	0.5538	0.105*	0.258 (10)
H10C	0.5030	0.8227	0.4283	0.105*	0.258 (10)
N1	0.8729 (2)	0.2761 (2)	0.23053 (18)	0.0502 (5)	
H1D	0.9620	0.2484	0.2073	0.060*	
O1	0.5162 (3)	0.35326 (19)	0.03480 (14)	0.0690 (5)	
O2	0.5283 (3)	0.56518 (19)	0.15897 (15)	0.0760 (6)	
H2A	0.5144	0.5865	0.0969	0.114*	
O3	1.0104 (4)	0.7427 (3)	0.4291 (3)	0.1314 (12)	
O4	0.6867 (3)	0.6538 (2)	0.42828 (16)	0.0787 (6)	
O5	0.1878 (2)	0.16074 (18)	0.18443 (16)	0.0603 (5)	
O6	0.2733 (2)	−0.03741 (17)	0.15535 (18)	0.0639 (5)	

### Atomic displacement parameters (Å^2^)

**Table:**

	*U*^11^	*U*^22^	*U*^33^	*U*^12^	*U*^13^	*U*^23^
C1	0.0385 (10)	0.0393 (10)	0.0487 (11)	0.0212 (8)	0.0147 (8)	0.0137 (8)
C2	0.0404 (10)	0.0340 (10)	0.0518 (12)	0.0200 (8)	0.0104 (8)	0.0091 (9)
C3	0.0447 (11)	0.0448 (11)	0.0502 (12)	0.0201 (9)	0.0089 (9)	0.0117 (9)
C4	0.0402 (11)	0.0529 (12)	0.0527 (12)	0.0210 (9)	0.0110 (9)	0.0169 (10)
C5	0.0443 (11)	0.0453 (11)	0.0554 (12)	0.0265 (9)	0.0186 (9)	0.0191 (9)
C6	0.0391 (10)	0.0404 (10)	0.0485 (11)	0.0221 (9)	0.0143 (8)	0.0159 (9)
C7	0.0421 (12)	0.0737 (17)	0.0839 (18)	0.0226 (12)	0.0055 (11)	0.0162 (14)
C8	0.0668 (16)	0.0540 (14)	0.0696 (16)	0.0268 (13)	−0.0015 (12)	−0.0009 (12)
C9	0.102 (4)	0.070 (3)	0.062 (2)	0.047 (3)	0.017 (2)	−0.004 (2)
C10	0.120 (4)	0.052 (2)	0.130 (5)	0.029 (3)	0.012 (4)	0.007 (3)
C11	0.0567 (14)	0.0494 (13)	0.0981 (19)	0.0345 (12)	0.0273 (13)	0.0212 (13)
C12	0.0423 (11)	0.0406 (11)	0.0555 (12)	0.0228 (9)	0.0169 (9)	0.0160 (9)
C13	0.0475 (13)	0.0486 (14)	0.124 (2)	0.0173 (11)	0.0306 (14)	0.0259 (15)
C14	0.0775 (19)	0.0460 (14)	0.148 (3)	0.0191 (14)	0.043 (2)	0.0279 (17)
N1	0.0374 (9)	0.0496 (10)	0.0691 (12)	0.0240 (8)	0.0191 (8)	0.0161 (9)
O1	0.1177 (15)	0.0498 (9)	0.0501 (10)	0.0486 (10)	0.0179 (9)	0.0143 (8)
O2	0.1359 (18)	0.0542 (10)	0.0573 (10)	0.0630 (12)	0.0200 (10)	0.0166 (8)
O3	0.0760 (15)	0.0632 (14)	0.184 (3)	0.0120 (12)	−0.0075 (16)	−0.0318 (16)
O4	0.0949 (14)	0.0754 (13)	0.0623 (11)	0.0545 (11)	0.0071 (10)	−0.0064 (9)
O5	0.0400 (8)	0.0500 (9)	0.0941 (13)	0.0261 (7)	0.0178 (8)	0.0163 (8)
O6	0.0432 (8)	0.0396 (8)	0.1140 (14)	0.0194 (7)	0.0259 (9)	0.0266 (9)

### Geometric parameters (Å, °)

**Table:**

C1—C6	1.510 (3)	C10—H10E	0.9600
C1—C2	1.524 (3)	C10—H10F	0.9600
C1—C3	1.528 (3)	C11—H11A	0.9600
C1—H1A	0.9800	C11—H11B	0.9600
C2—O1	1.217 (3)	C11—H11C	0.9600
C2—O2	1.304 (2)	C12—O5	1.222 (2)
C3—C4	1.357 (3)	C12—O6	1.332 (2)
C3—C8	1.472 (3)	C13—O6	1.462 (3)
C4—N1	1.383 (3)	C13—C14	1.483 (4)
C4—C7	1.505 (3)	C13—H13A	0.9700
C5—C6	1.363 (3)	C13—H13B	0.9700
C5—N1	1.378 (3)	C14—H14A	0.9600
C5—C11	1.508 (3)	C14—H14B	0.9600
C6—C12	1.465 (3)	C14—H14C	0.9600
C7—H7A	0.9600	C9'—C10'	1.499 (15)
C7—H7B	0.9600	C9'—O4	1.649 (12)
C7—H7C	0.9600	C9'—H9'A	0.9700
C8—O3	1.204 (3)	C9'—H9'B	0.9700
C8—O4	1.348 (3)	C10'—H10A	0.9600
C9—O4	1.432 (4)	C10'—H10B	0.9600
C9—C10	1.461 (8)	C10'—H10C	0.9600
C9—H9A	0.9700	N1—H1D	0.8600
C9—H9B	0.9700	O2—H2A	0.8200
C10—H10D	0.9600		
			
C6—C1—C2	111.10 (16)	H10E—C10—H10F	109.5
C6—C1—C3	111.42 (16)	C5—C11—H11A	109.5
C2—C1—C3	108.83 (16)	C5—C11—H11B	109.5
C6—C1—H1A	108.5	H11A—C11—H11B	109.5
C2—C1—H1A	108.5	C5—C11—H11C	109.5
C3—C1—H1A	108.5	H11A—C11—H11C	109.5
O1—C2—O2	122.4 (2)	H11B—C11—H11C	109.5
O1—C2—C1	122.99 (17)	O5—C12—O6	122.05 (19)
O2—C2—C1	114.51 (17)	O5—C12—C6	122.53 (19)
C4—C3—C8	121.5 (2)	O6—C12—C6	115.42 (17)
C4—C3—C1	119.72 (19)	O6—C13—C14	106.9 (2)
C8—C3—C1	118.72 (19)	O6—C13—H13A	110.3
C3—C4—N1	119.07 (18)	C14—C13—H13A	110.3
C3—C4—C7	127.0 (2)	O6—C13—H13B	110.3
N1—C4—C7	113.86 (19)	C14—C13—H13B	110.3
C6—C5—N1	118.93 (18)	H13A—C13—H13B	108.6
C6—C5—C11	127.8 (2)	C13—C14—H14A	109.5
N1—C5—C11	113.28 (17)	C13—C14—H14B	109.5
C5—C6—C12	125.35 (19)	H14A—C14—H14B	109.5
C5—C6—C1	120.07 (18)	C13—C14—H14C	109.5
C12—C6—C1	114.31 (16)	H14A—C14—H14C	109.5
C4—C7—H7A	109.5	H14B—C14—H14C	109.5
C4—C7—H7B	109.5	C10'—C9'—O4	97.3 (9)
H7A—C7—H7B	109.5	C10'—C9'—H9'A	112.3
C4—C7—H7C	109.5	O4—C9'—H9'A	112.3
H7A—C7—H7C	109.5	C10'—C9'—H9'B	112.3
H7B—C7—H7C	109.5	O4—C9'—H9'B	112.3
O3—C8—O4	122.4 (3)	H9'A—C9'—H9'B	109.9
O3—C8—C3	125.8 (3)	C9'—C10'—H10A	109.5
O4—C8—C3	111.8 (2)	C9'—C10'—H10B	109.5
O4—C9—C10	107.7 (4)	H10A—C10'—H10B	109.5
O4—C9—H9A	110.2	C9'—C10'—H10C	109.5
C10—C9—H9A	110.2	H10A—C10'—H10C	109.5
O4—C9—H9B	110.2	H10B—C10'—H10C	109.5
C10—C9—H9B	110.2	C5—N1—C4	123.75 (16)
H9A—C9—H9B	108.5	C5—N1—H1D	118.1
C9—C10—H10D	109.5	C4—N1—H1D	118.1
C9—C10—H10E	109.5	C2—O2—H2A	109.5
H10D—C10—H10E	109.5	C8—O4—C9	121.9 (3)
C9—C10—H10F	109.5	C8—O4—C9'	97.6 (5)
H10D—C10—H10F	109.5	C12—O6—C13	117.96 (17)
			
C6—C1—C2—O1	−17.2 (3)	C1—C3—C8—O3	−158.8 (3)
C3—C1—C2—O1	105.9 (2)	C4—C3—C8—O4	−160.1 (2)
C6—C1—C2—O2	166.01 (18)	C1—C3—C8—O4	22.5 (3)
C3—C1—C2—O2	−70.9 (2)	C5—C6—C12—O5	−172.2 (2)
C6—C1—C3—C4	25.8 (3)	C1—C6—C12—O5	1.8 (3)
C2—C1—C3—C4	−97.1 (2)	C5—C6—C12—O6	8.0 (3)
C6—C1—C3—C8	−156.7 (2)	C1—C6—C12—O6	−177.98 (17)
C2—C1—C3—C8	80.4 (2)	C6—C5—N1—C4	13.9 (3)
C8—C3—C4—N1	175.1 (2)	C11—C5—N1—C4	−166.0 (2)
C1—C3—C4—N1	−7.5 (3)	C3—C4—N1—C5	−14.1 (3)
C8—C3—C4—C7	−2.6 (4)	C7—C4—N1—C5	163.9 (2)
C1—C3—C4—C7	174.8 (2)	O3—C8—O4—C9	0.8 (5)
N1—C5—C6—C12	−178.35 (18)	C3—C8—O4—C9	179.6 (3)
C11—C5—C6—C12	1.5 (4)	O3—C8—O4—C9'	15.3 (6)
N1—C5—C6—C1	8.0 (3)	C3—C8—O4—C9'	−165.9 (5)
C11—C5—C6—C1	−172.2 (2)	C10—C9—O4—C8	89.7 (5)
C2—C1—C6—C5	95.5 (2)	C10'—C9'—O4—C8	172.6 (8)
C3—C1—C6—C5	−26.0 (3)	O5—C12—O6—C13	2.0 (3)
C2—C1—C6—C12	−78.8 (2)	C6—C12—O6—C13	−178.2 (2)
C3—C1—C6—C12	159.62 (17)	C14—C13—O6—C12	173.7 (2)
C4—C3—C8—O3	18.6 (5)		

### Hydrogen-bond geometry (Å, °)

**Table:**

*D*—H···*A*	*D*—H	H···*A*	*D*···*A*	*D*—H···*A*
N1—H1D···O5^i^	0.86	2.17	3.018 (2)	167
O2—H2A···O1^ii^	0.82	1.82	2.641 (2)	176

Symmetry codes: (i) *x*+1, *y*, *z*; (ii) −*x*+1, −*y*+1, −*z*.
